# Exploring Competencies and Skills of Australian Rural Psychologists

**DOI:** 10.1111/ajr.70228

**Published:** 2026-07-01

**Authors:** Margot P. Moody, Natasha M. Loi, Adam J. Rock, Kim J. Usher, Kylie Rice

**Affiliations:** ^1^ School of Psychology University of New England Armidale New South Wales Australia; ^2^ School of Health University of New England Armidale New South Wales Australia

**Keywords:** competencies, generalist, psychologist, rural, skillset

## Abstract

**Background:**

The ratio of psychologists to the population is vastly lower in rural communities compared to urban areas, and more clinicians are critically needed in rural Australia. Other health disciplines have implemented generalist frameworks and training to encourage and prepare clinicians to remain in rural practice. Research often references rural psychology practice in conjunction with the broader allied health discipline.

**Objective:**

Though prior research has identified skills needed for rural psychology practice, further research is needed to expand and update the current understanding of rural psychology competencies. Therefore, the current study explored the competencies and skills used by rural psychologists.

**Methods:**

Qualitative interviews with 15 rural psychologists from across Australia were conducted and analysed using a reflexive thematic approach.

**Results:**

Participants spoke of the unique skills required to manage the challenges of rural practice, identifying eight thematic competencies: *clinical skills, networking, cultural responsiveness, practitioner wellbeing, ethics, risk, professional development* and *telehealth.*

**Conclusion:**

Themes highlighted the need for a discipline‐specific approach to supporting rural psychologists in practice. Applications of this research, particularly towards supporting and retaining psychologists in rural Australia, are discussed.

## Key Points

**What Is Already Known About This Topic:**

- Psychologists working in rural Australia face challenges such as high workloads, personal and professional isolation, and increased ethical challenges, impacting recruitment and retention efforts.
- Various health disciplines have employed rural‐specific competency and training frameworks to ensure clinicians are prepared for rural health care; however, none as yet exist specific to psychology.
- Prior research suggests the need for rural‐specific skills and competencies for psychologists working in such communities.

**What This Topic Adds:**

- Rural psychologists employ a wide variety of skills, which align with their broader psychological competencies; however, the rural context impacts how these skills are operationalised.
- Comparisons to broader allied health rural frameworks highlight the uniqueness of the psychology profession when working with rural communities.
- Psychologists wishing to practice in rural areas would benefit from rural‐specific training and support.

## 1 Introduction

Rural communities face an ongoing struggle to access mental health services, with mental health needs far outweighing the availability of relevant services [1, 2]. Though rural people have been found to have more positive attitudes to help‐seeking than their urban counterparts, they also experience greater psychological distress, with limited service availability identified as a prominent barrier to receiving mental health support [3]. Service shortages are not restricted to psychology and mental health professions. Recruitment challenges and strategies for rural allied health staff, nurses and medical practitioners, particularly, have also been widely explored in both the literature and health policy [4, 5, 6].

In the Australian context, rural health recruitment challenges are underscored by a maldistributed workforce, due in part to the vast geographical distances between communities, yet highly populated capital cities [7]. For example, though recruiting psychologists is a national challenge [8], in 2024, of the 36 871 psychologists registered and employed in Australia, 1397 were based in outer regional areas, while only 149 worked in remote areas [9]. The mental health workforce in remote New South Wales represented a significantly decreased ratio of 26 psychologists per 100 000 people, compared to 125 psychologists per 100 000 nationally, with similar ratios evident across remoteness areas in all Australian states [10]. Workforce shortages in rural communities are further complicated by retention difficulties, with rural allied health professionals, including psychologists, typically having shorter employment tenures on average, compared to urban health professionals [4].

Rural mental health clinicians face challenges related to dual relationships, high workloads and isolation [11]. For psychologists specifically, these challenges have impacts on both a personal and professional level, across community, occupation career and training domains [12]. Rural psychology work requires unique knowledge and skills [13], however, clinicians are not often trained to manage rural‐specific challenges. Research is clearly needed to identify strategies that better train and support rural clinicians and ultimately improve service access for rural people.

### 1.1 Rural Recruitment Strategies

Internationally, the most effective predictors of the recruitment and retention of rural health practitioners have been identified to be rural origin and opportunities for rural immersion [6, 14, 15]. For both urban and rural trainees alike, rural placements and curriculum have been found to increase the likelihood of students remaining in rural practice [14]. This approach provides trainees the opportunity to experience a rural lifestyle while gaining the clinical experience and skills necessary to work effectively in rural practice [16, 17, 18]. Other strategies such as bonded placements (in exchange for visa waivers or reduced loan repayments, for instance) and financial incentives have shown varying effectiveness in helping retain practitioners in rural areas [6, 15]. In Australia, the government has adopted a range of initiatives to grow the rural health workforce. The Stronger Rural Health Strategy released in 2018 includes initiatives such as rural‐specific training and placements, increased funding for rural health services, and financial incentives [5]. Early evaluations of this strategy suggest that increasing training opportunities in rural and remote areas via the Rural Health Multidisciplinary Training Programme have resulted in positive outcomes for the rural workforce [19].

### 1.2 Rural Generalism

Rural health work can be challenging, requiring health professionals to possess a broad set of skills in order to treat the diverse needs of a community competently [20]. To this end, many health disciplines have adopted the term ‘rural generalist’ (e.g., general practitioners (GPs) [21], nurses [22]), defined as a clinician with the recognised skills and qualifications to provide care across health care settings in rural/remote or regional communities [23]. In training rural generalists and to further assist with rural preparedness, health disciplines have developed specific rural generalist programmes and pathways, including training and support for rural competencies [20, 21].

Specific to Australia, these programmes have been developed for GPs [21], nurses [22] and allied health professionals [20]. The National Rural Generalist Pathway is the latest evolution in a series of initiatives for rural GPs, designed to provide clear training and support for practitioners to better service rural communities [21]. Rural nurses have also been recognised for their unique skills, given they are often required to fill the positions of medical and other health professionals whilst also using a broad set of skills within their own discipline [24]. The National Rural and Remote Nursing Generalist Framework has been developed to help inform training and support for nurses in rural practice [25], with assessments regarding the efficacy and utility of this framework ongoing.

For allied health professionals, the recently developed Allied Health Rural Generalist Programme offers a tertiary level education course covering service delivery and clinical skills across health disciplines, alongside other workplace and training support [20, 26]. The programme includes six core modules (project management skills, rural and remote community context, partnering with Aboriginal and Torres Strait Islander health consumers, rural and remote organisational context, strategies for rural and remote service delivery and quality improvement) and an additional six modules from four focus areas (ages and stages across the lifespan, managing health conditions, clinical skills and service‐specific clinical skills) relevant to each profession [26]. Evaluations of the programme suggest that it has the potential to increase tenure intentions and reduce turnover in participating clinicians, with students reporting an increase in confidence and competence in rural practice skills [20, 27, 28]. However, as the programme is broadly targeted for all allied health professionals, discipline‐specific content and alignment to professional competencies is minimal [20]. Furthermore, psychologists' participation in the programme appears low, taking up just 3% of enrolments in 2017–2019 [20].

Understanding the efficacy of such strategies and programmes targeting allied health professionals is further complicated given the inconsistency in the literature regarding whether to treat these clinicians as a group or as specific disciplines [4]. Though the rural allied health workforce is inflicted by similar broad recruitment challenges, the vast differences in scope, approach and the contexts in which clinicians operate suggest there are likely to be discipline‐specific challenges as well [29, 30]. Further, treating allied health professionals as a homogenous group can hide workforce shortages, as some professions (such as pharmacy and physiotherapy) have greater representation in rural areas compared to other professions (such as psychology) [31]. To this end, rural challenges unique to individual professions, such as ethical challenges and dual relationships for psychologists, may not be adequately addressed in broader allied health programmes.

### 1.3 The Rural Psychologist

Psychology in Australia is a competency‐based profession. Though one can possess knowledge or skills relevant to their role, competencies refer to the actions or behaviours using combined knowledge, skills and values required to deliver safe and effective practice [32]. Australian clinicians are required to abide by professional practice standards as a requirement of their professional registration [33]. These standards, which were recently updated and effective as of December 2025, are inclusive of the Professional Competencies for Psychologists and the Code of Conduct [33]. Both standards detail the expectations of safe and effective psychological practice across the broad scope of the profession, and inform education and training practices for all psychologists. To support these updated standards, the Australian Psychological Society (APS) provided revised professional practice guidelines specific to providing services in rural and remote environments [34]. Though they do not speak to specific professional competency requirements, the guidelines aim to support rural practitioners to interpret and comply with their professional standards, given the challenges of rural practice [34].

Currently, the Allied Health Rural Generalist Programme [26] is the only known rural programme applicable to psychologists in Australia, with no other specific framework or training available. Development of a rural psychology programme or framework first requires a detailed understanding of the competencies required of a rural psychologist [35]. This in turn will better prepare psychologists for rural practice. Rural people have identified the desire for psychologists to be culturally competent; that is, proficient in rural‐specific challenges and culture [36]. Prior research by Sutherland and Chur‐Hansen [13] explored the knowledge, skills and attitudes used by rural and remote psychologists, with participants reporting competencies relating to managing both professional isolation and the rural context of their roles, alongside necessary personality traits. Notably, both the authors and participants acknowledged the need for rural‐specific training and placements to best develop the skills required for rural practice [13]. Follow up research by Sutherland et al. [37], reported that Fly‐In‐Fly‐Out (FIFO) practitioners require similar skills to rural‐based practitioners, in addition to unique support needs. However, although Sutherland and Chur‐Hansen [13] identified seven rural competencies, additional research is needed to further amalgamate the broader knowledge and skillset. Given the significant changes to competency requirements and professional practice standards required of all psychologists in recent years [33], an updated review of rural specific psychology competencies is required. The current study aimed to consolidate and advance the previous research by Sutherland and Chur‐Hansen [13] and provide an updated understanding of the specific competencies and skills used by rural psychologists.

## 2 Method

### 2.1 Participants

A total of 15 participants completed interviews as part of this study. They included registered psychologists, including those with and without endorsements, with current or prior experience in rural mental health. Participants were recruited across Australia via professional networks. Though prior research has recognised differences in skills and experiences between FIFO practitioners and those who live and work in rural communities [37], all rural practitioners were invited to participate in order to provide a comprehensive understanding of the various competencies used by all rural modalities. Due to the identifying nature of qualitative and small sample research, demographic information collected for this study was limited to gender, years of experience and work structure. Of the sample, 73.3% were female and 26.6% were male. Participants reported an average of 10.4 years of psychological practice, of which 8.4 years was in rural practice. Ten participants reported living in the same community they supported, while the remainder of participants reported a combination of telehealth or fly‐in‐fly‐out modalities.

### 2.2 Procedure

Participants attended individual semi‐structured interviews with the first author via Zoom. At the beginning of the interview, participants were asked to provide some brief demographic information and informed consent. Interviews were audio recorded and ran approximately 1–2 h each. Following the interviews, recordings were transcribed verbatim. Ethical approval was provided by the University's Human Research Ethics Committee.

### 2.3 Materials

#### 2.3.1 Interview Questions

Participants were asked to describe the skills, competencies and experiences related to rural psychology practice, including their mode of delivery (‘What primary mode/s of service delivery do you use? Does this require any specific skills?’), clinical work (‘What theoretical approach do you use the most and why?’), cultural skills (‘What different cultural groups do you work with?’), ethics (‘What type of ethical issues do you encounter in your work? How do you manage these?’), non‐clinical tasks (‘What non‐clinical tasks do you perform in your work? What skills help you perform these tasks?’) and training and supervision (‘How do you access supervision/professional development?’). At the end of the interview, participants were also asked to consider what skills or competencies should be included in a model of rural psychology competencies.

### 2.4 Qualitative Analysis

Themes were generated using inductive reflexive thematic analysis, adopting an experiential orientation [38]. Though previous research has identified some rural psychology skills and competencies [13, 37], these have yet to be recognised as standardised rural psychological practices. Therefore, the analytical approach used ensured participants' voices and experiences guided findings, while allowing for systematic but flexible rigorous exploration of the data. Likewise, Australian‐specific terminology was retained, including the use of ‘Aboriginal and Torres Strait Islander people’ to respectfully refer to Indigenous Australians, consistent with the language used within the Psychology Board of Australia's competency documents [33].

Deidentified interview transcripts were imported to nVivo and analysed using Braun and Clarke [39] six‐phase approach, with analysis completed primarily by the first author (M.M.). Following the initial interview, the research team reviewed the transcript to check appropriate procedures were being followed. Initial codes and a thematic map were also created to ensure questions and procedures were generating relevant data. Further interviews were then conducted until sufficient depth and richness of information was achieved [40]. Given the specificity of the research question and the detailed, relevant data generated from participants' responses [38, 40], a final sample size of 15 was deemed adequate to address the research questions. Once interviews were completed, all transcripts were reviewed against audio recordings to assess the accuracy of the transcripts and to assist with data immersion. Recordings were then continuously reviewed throughout the analysis process. Transcripts were analysed using open‐ended coding, allowing for a flexible and data‐driven interpretation of responses. Codes were then reviewed and analysed to create initial themes. Codes and themes were reviewed and refined in a cyclical process, with iterations continuously shared and discussed with the wider research team (N.L., A.R., K.U., K.R.) until the final themes were identified and defined.

Throughout analysis, and indeed the entire research project, a reflexive approach was taken. This approach requires researchers to critically examine their assumptions, values and personal and professional backgrounds, and how these factors may impact the research process [38]. The research team had prior experience in rural psychology and was conscious of how this experience could impact analysis. This positionality meant that the team brought particular assumptions regarding rural practice, including service access challenges and dual relationships, likely impacting the way in which interviews were conducted, transcripts coded and themes created. To this end, researchers' experiences were openly discussed while reviewing the themes to ensure subjectivity was assisting, rather than impeding, interpretation of participants' responses. Likewise, journalling and self‐reflection were used by the first author throughout the project.

## 3 Results

**FIGURE 1:**
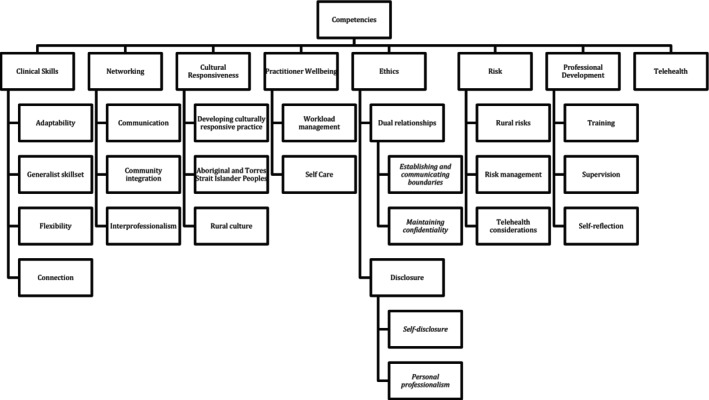
Competencies used as reported by Australian rural psychologists.

Participants' responses revealed eight themes relating to rural‐specific psychology competencies. As depicted in Figure 1, themes included *clinical skills, networking, cultural responsiveness, practitioner wellbeing, ethics, risk, professional development* and *telehealth. Clinical skills* encompassed skills relevant to clinical practice that aided in providing evidence‐based care in a way that was accessible to the rural community and clientele such as adaptability, flexibility, facilitating connection and working as a generalist. *Networking* acknowledged the need to have a vast range of communication and networking skills in order to both engage with clients and the community and avoid isolation. *Cultural responsiveness* included the appreciation of the unique aspects of both Aboriginal and Torres Strait Islander peoples and rural culture at the community level and in the broader cultural context. Participants spoke of developing an understanding of various cultures and adapting skills to best connect with individuals and their community while also being mindful of their own biases and cultural responsiveness as an ongoing practice.

*Practitioner wellbeing* recognised the potential impacts to wellbeing when working rurally, with participants identifying processes and strategies to support self‐care and mitigate against burnout. The *ethics* theme involved the understanding of common ethical challenges related to working in close proximity to clients and developing a sense of comfort with this. Participants identified processes and strategies to best manage the impacts of ethical challenges, particularly those relevant to dual relationships and disclosure. *Risk* included the unique risks associated with rural culture and communities and the skills required to best manage these. Participants working via telehealth or living outside of the communities they supported identified additional skills required to manage risks. *Professional Development* spoke to the processes and skills of proactively identifying the need to seek further training or support, particularly in the context of the challenges associated with working rurally, such as professional isolation. Lastly, all participants spoke of using telehealth at some point in their roles, as identified in the *telehealth* theme. This theme was inclusive of all skills required to best engage clients and adapt therapeutic approaches for telehealth delivery. See Table 1 for theme and sub‐theme definitions.

**TABLE 1:** Competency themes and sub‐themes.

Themes/sub‐themes	Definition	Example quote
*Clinical skills*		
Adaptability	Adapting how participants deliver a particular treatment or modality to best fit with the client and service delivery method (and rural context/environment), while remaining evidence‐based and relevant to that person.	‘I just had to change around the way that I was session planning; I'd hold back on doing a fair bit of kind of exposure protocols because I just couldn't fit it in… I didn't want to kind of lead people down this path of doing unsafe practice. We'll do this really intensive trauma work and then I won't see you in person for a month’ (Participant F)
Generalist skillset	Due to limited availability of referral options, needing to have diverse knowledge and skillset to work with a range of ages and presentations, while also working within limits of competency.	‘Having that comfortability with working across the entire age and cultural range of people’ (Participant M)
Flexibility	Being flexible with caseload, scheduling, referral acceptance, or role requirements in order to meet need and work within client, community and cultural contexts.	‘It's not always going to be like a We have a 50 min session at 9 o'clock…just being open to having to kind of change things up…there might be times where a family or a parent can't get somewhere’ (Participant B)
Connection	Developing a connection with clients to facilitate trust and rapport by being genuine and relatable, particularly to align with rural values and to support those who had not sought help before.	‘Being relatable is probably the biggest thing, the biggest benefit that will get you in with the communities’ (Participant K)
*Networking*		
Communication	The need to have clear communications with clients, the broader community, and other health professionals, though this may be more relaxed, and relevant to the rural context. Communication is vital for building connections and coordinating care with limited referral options.	‘When I think about working as a rural psych, the first thing that comes to my head is communication’ (Participant A)
Community integration	Both rural culture and context mean practitioners are more visible in the community. This requires them to work at both an individual and community level; integrating oneself as a member of the community to build trust, who can also be seen as knowledgeable and credible.	‘When you have shared experiences like getting clinicians out, actually working rural, you can relate more strongly to things that they're going through’ (Participant O)
Interprofessionalism	Building relationships with other health professionals and community members/stakeholders to assist with consistency of care for clients (given limited referral options) and avoid professional isolation. It encompasses the skills needed when working within a multidisciplinary team, often as the only psychologist in the team or community, and understanding how the role of psychologist fits in that team.	‘Your ability to make connections with other agencies because you are limited by you've kind of got to figure it out and. Be pretty good at connecting in with what limited things are available’ (Participant B)
*Cultural responsiveness*		
Developing culturally responsive practice	The broader skills required when working with culturally diverse clients, given the diverse demographic and clinical presentations that rural practitioners may work with. Specifically, understanding that clients come from different cultural backgrounds and contexts, developing knowledge and awareness of different cultures, being mindful of one's own biases and gaps in knowledge, and reflecting that this is an ongoing skill.	‘Being aware of the different cultures and how they view the world that we live in. And I think being humble in learning from those families that come from a different cultural perspective. And being able to accept that I don't know everything’ (Participant N)
Aboriginal and Torres Strait Islander Peoples	Developing an understanding of Aboriginal and Torres Strait Islander culture, knowledge of skills and therapeutic approaches that may support work with clients. Increasing knowledge and awareness of local Aboriginal and Torres Strait Islander culture while being aware of limitations of knowledge.	‘I think some of it (is) skills and some of it is knowledge. So, for example, learning the names for different things around the area, different landmarks and what they are in the local language and things like that, and learning people who are, you know, elders in the community and understanding the impact when they're unwell, or if they pass, sorry business comes up. So, there's been an element of learning as well as skill change. If that makes sense’ (Participant D)
Rural culture	Developing an understanding of rural culture and how it might impact therapeutic approaches, including how rural people view services and professionals, how they engage in support, and what sort of issues may impact them. Understanding that each community has its own systems, culture and history that shape how they interact with services and each other.	‘How rural people see the world compared to urban people, how they see systems and professionals can vary, so I think…rurality and country culture can be treated as a cultural issue in its own right’ (Participant G)
*Practitioner wellbeing*		
Workload management	Skills to manage caseload and work schedules, including implementing boundaries, to avoid burnout and maintain well‐being in the context of constant referrals and work demands.	‘Time management. Trying to juggle an increasingly large caseload of clients is difficult with all the meetings and the case notes and you know, NDIS applications and everything that comes with it’ (Participant J)
Self‐Care	Developing practices to ensure self‐care, particularly given the rural context of isolation, limited personal resources/services and increased risk of burnout for rural practitioners.	‘Self‐care can be harder for people in rural areas when there isn't a lot of specific activities available or there aren't as many’ (Participant E)
*Ethics*		
Dual relationships	Given the small and interconnected nature of many rural communities, accepting dual relationships as part of the role and developing processes to manage against ethical challenges that arise from such relationships.	‘Sitting with discomfort. Because there are going to be layers of that. You know you are going to be talking to Bill, and Bill is going to be maybe living two blocks away from you. Not that he will know that, but you'll know that from looking at his residential address. Bill might even be like the Under 5 soccer coach that you're just about to enrol your son into soccer. You got to sit with that, and it can be incredibly awkward. Or you can just roll with it’ (Participant E)
Establishing and communicating boundaries	Creating and maintaining boundaries when accepting referrals, seeing clients, and engaging with the community.	‘You just need to kind of keep having that open communication about how those boundaries are feeling for each of you, it's not just a one‐off conversation’ (Participant A)
Maintaining confidentiality	Awareness that psychologists also engage in multiple roles and ensuring information remains confidential to relevant sources.	‘You gotta be careful about using the information that you might have. That sort of personal information that you might have through other sources. But in assessing safety, you know, I think. It can be useful to use that information’ (Participant H)
*Disclosure*		
Self‐disclosure	Understanding the potential need to share more of oneself with clients and the wider community to build trust and align with rural values, while also ensuring ethical boundaries.	‘Self‐disclosure and like sharing more of your story and kind of more of your not necessarily life experience, but like who you are and where you belong. Having to tell a little bit of your own story to get people to open up about their story’ (Participant F)
Personal professionalism	Understanding that in small communities, participants identities are more visible. Being mindful of one's own personal presence in the community; how personal and professional life are intertwined. Managing personal interactions in the community while also demonstrating professionalism and respect for the profession.	‘Your values as a psychologist, having to overlap very much with your values as a person, because people are going to see the way you (are) behaving community’ (Participant D)
*Risk*		
Rural risks	Knowledge of how risks may present differently in rural areas and specific local communities.	‘There's a lot of slightly risky sports that are normalized here, like dirt bike riding and things like that. I think you need to factor in when you're considering someone's risk as well’ (Participant D)
Risk management	Utilising knowledge of rural risks to adapt and implement assessments and safety plans for the rural context. Capacity to work with risks in culturally relevant risk management protocols.	‘Competency to deal with, to work with suicidality, and work helpfully and constructively with that psychologically…especially without having access to ready services to back you up necessarily, you may be quite isolated. How do you work with suicidality safely?’ (Participant G)
Telehealth considerations	Applying the knowledge of rural risks and risk management protocols to the telehealth environment. Developing processes to ensure safety during and after telehealth consults, including the ability to acquire local contacts and networks.	‘I think part of the challenge in telehealth is that I might have good awareness of my local supports and services and options, but I may not have that same degree of awareness for somebody who is 500 kilometres away. So again, it's a bit of if you're going to work in those spaces, you. You probably need to be aware of what some local supports are’ (Participant I)
*Professional development*		
Training	Prioritising and maintaining relevant professional development given the limited training opportunities outside of urban areas; sourcing training opportunities to upskill and support the generalist skillset.	‘Having a bit more of a structured plan about professional development’ (Participant N)
Supervision	Developing resources and networks to access supervision when needed and avoid professional isolation (inclusive of both peer and professional supervision).	‘I've been really deliberate in building a supervision network’ (Participant I)
Self‐Reflection	Ability to continuously engage in reflective practice, particularly when managing risk, ethical or cultural issues pertinent to rural practice.	‘Okay, what can I do differently and not getting stuck in your own practice and being open to being wrong and just being like, yeah, maybe I was doing a really shit job. Is probably how I would describe it’ (Participant B)
*Telehealth*		
Telehealth	All participants spoke of utilising telehealth at some point in their work with rural communities. This competency encompasses all skills required to deliver services via telehealth, including knowledge of digital modalities, platforms and strategies to best facilitate sessions, adapting resources to be used digitally, and managing distractions and obstacles for both clinician and client.	‘The basic technical stuff, like how to support people. But if it's freezing and dropping out, then just straight away getting people to connect by phone and we can do the, you know, the talking part by phone, but then still have the screen… so that it's not disrupted’ (Participant H)

## 4 Discussion

The current study aimed to identify the competencies and skills used by rural psychologists in Australia. Psychologists living and working in regional, rural and remote communities, as well as those in fly‐in‐fly‐out and telehealth roles, participated in online qualitative interviews. Participants identified eight thematic competencies used in rural practice: *clinical skills, networking, cultural responsiveness, practitioner wellbeing, ethics, risk, professional development* and *telehealth*. Excluding the *risk* and *telehealth* themes, the majority of the competencies were consistent with Sutherland and Chur‐Hansen's [13] research.

### 4.1 Alignment With Broader Professional Competencies

Though some skills identified within thematic competences appear unique to the rural profession (*community integration, rural risks, rural culture* and *self‐disclosure*), the competencies reported in the current study mostly align with the Professional Competencies for Psychologists, as outlined by the Australian Health Practitioner Agency [33]. It is not surprising that many of these competencies were identified in participants' responses, such as the need to practice ethically and professionally, maintain competence, seek supervision, manage risk, practice self‐care and be culturally responsive. Though these competencies are relevant within the scope of professional psychologists regardless of location, participants' responses indicated that the rural context impacted how they were utilised. For example, telehealth usage has increased across geographic locations since the COVID‐19 pandemic [41, 42], with guidelines relevant to both rural and urban psychologists in practice [43]. The inclusion of the *telehealth* theme as a skill specific for rural clinicians highlights the increased reliance on this modality to improve service availability in isolated areas.

Likewise, all Australian psychologists are required to be competent in working across the lifespan, in a variety of contexts and with range of assessment and treatment methods [33]. Despite this, many clinicians choose to specialise in specific age groups, presentations, or contexts [44]. In rural areas characterised by reduced services and limited clinician capacity, it is well established that health practitioners are frequently required to work as generalists [20, 45, 46]. However, less research has focused on how psychologists operate as generalists in rural areas. This broader skillset, alongside skills such as *adaptability* and *flexibility*, were identified by participants in the *clinical skills* theme, and emphasised the importance of clinician's ensuring their broader clinical competencies were tailored to the needs of community. Standard skills such as good communication and professionalism were similarly contextualised for the rural context. In lieu of available and reliable referral pathways, participants acknowledged the importance of proactively engaging with other health professionals and the wider community, in order to better facilitate care and avoid isolation. As identified in the *networking* theme, the lack of anonymity and use of informal communication networks (information sharing through relationships rather than via formal channels, e.g., word of mouth) in rural areas [47], required participants to adjust how they interacted and engaged with clients, community members and stakeholders.

The *cultural responsiveness* theme, particularly regarding working with Aboriginal and Torres Strait Islander Peoples, is a necessary competency for all psychologists regardless of location. Recent updates to the professional competencies and standards required of Australian psychologists have included a greater emphasis on cultural safety, cultural responsiveness and working with diverse groups [33]. As reflected in the participants' responses, cultural responsiveness is especially important for those working in rural and remote communities, given the higher proportion of Aboriginal and Torres Strait Islander Peoples in these communities [48]. In both the established literature and the current study, the need for clinicians to be culturally responsive extends to the unique rural context of clients and their community [45, 49]. Though rural culture is not explicitly mentioned in the recently released APS guidelines for rural and remote practice, the importance of cultural responsiveness and reflexivity, with respect to both Aboriginal and Torres Strait Islander Peoples and diverse client groups, is highlighted [34]. Rural mental health consumers' endorsement of mental health clinicians with rural knowledge who prioritise connection with their clients further emphasises the importance of these cultural competencies [36].

Ethical practice is a core tenet of safe and effective service delivery for Australian psychologists [33]. Due to the limited available services and close networks in rural areas, managing ethical dilemmas is a common challenge for many rural psychologists [13], as identified by participants in the *ethics* theme. Wherein urban areas, an ethical dilemma such as a dual relationship can be managed by referring a client to another service, rural clinicians have to consider the ethical implications of engaging in such relationships or potentially leave the client unsupported. In the current study, participants spoke of needing a strong ethical base to navigate such dilemmas, with dual relationships and personal disclosure common occurrences in both their personal and professional lives. A primary aim of the APS rural and remote practice guidelines is to support rural clinicians interpret and comply with their ethical and professional obligations as per the Code of Conduct for Psychologists [33, 34]. Many of these guidelines relate to competencies and skills identified in the current study, including networking, managing wellbeing, delivering telehealth and the aforementioned cultural responsiveness. In particular, guidelines specific to ethical challenges (e.g., boundaries, multiple relationships, managing confidentiality, and being mindful of personal and professional identities within the community), closely align with the *ethics* competency and accompanying skills described by participants, further highlighting how the rural context impacts ethical behaviours and decision making.

Though *professional development* is a requirement of registration for all Australian psychologists, participants highlighted how the geographic and professional isolation of rural practice requires a proactive approach to maintaining training and supervision. Previous research has acknowledged the paradox of managing professional development as a rural clinician [12]. For example, though clinicians can consider a wide variety of training opportunities due to their diverse client range, access to in‐person training and supervision can be challenging [12]. The current study highlights how practitioners plan and navigate their professional development needs to avoid such access challenges. Similarly, the *wellbeing* theme articulated how participants needed to be mindful of protecting themselves against burnout and moral injury. Burnout is a common side effect of the demanding and complex workloads faced by many psychologists and has been found to be a predictor of depression in clinicians [50]. Psychologists are encouraged to prioritise self‐care and minimise lifestyle and work stressors [51], with the updated professional competencies for psychologists reflecting greater emphasis on the importance of self‐care [33]. However, as participants in the current study noted, isolation and reduced access to services can make self‐care for rural practitioners difficult. Though the country lifestyle has been found to be an attractor to rural practice [12, 52], such a lifestyle requires clinicians to set boundaries and develop a practical approach to self‐care.

The *risk* theme, though recognised as a professional competency for psychologists and indeed in rural health literature more broadly [33, 53, 54, 55], is not referenced as an explicit skill required for either psychologists or allied health rural practitioners, nor is it directly acknowledged in the APS guidelines specific to rural and remote settings [34]. However, the lack of consideration for risk management as a skill is concerning, given the higher rates of suicide in rural communities, limited access to other support services, and increased likelihood of clinicians working with high‐risk clients [54, 56]. Contextual factors such as long work hours, access to lethal means, and the effects of natural disasters have been found to impact suicide and mental health in farming communities [57], with Kunde, Kõlves [58] positing that considering sociocultural factors in suicide prevention is important for rural communities. The literature has advocated for the need to address risk and suicidal behaviours, particularly for those in rural areas, differently [59, 60], and participants in the current study identified some targets for intervention.

### 4.2 Rural Allied Health Competency Frameworks

In the absence of a psychology‐specific rural framework, the current results were also examined in the context of broader rural allied health competency frameworks. Participants' responses were mostly consistent with competencies identified in a competency framework specifically created for rural and remote senior allied health professionals in Western Australia [61]. Responses similarly aligned with the Allied Health Rural Generalist Education Framework [55]. This framework informs the Rural Generalist Programme and acknowledges the rural and remote context in various professional skills, such as conducting ethical practice, accessing education and supervision, delivering telehealth, networking and adapting service delivery within each discipline [55]. However, both frameworks are also inclusive of broader professional and managerial skills and those unique to the health systems from which they were created; skills which were not identified in the current study. Likewise, ethical practice is included in both frameworks, though not to the depth that was discussed by participants. This is not to suggest rural allied health frameworks do not offer utility to psychologists. Rather, the results emphasise the need to acknowledge the uniqueness of rural psychological practice that is not reflected in the current rural generalist discourse.

### 4.3 Clinical Implications

The themes identified by participants highlight that there are unique competencies and skills required for psychologists to practice rurally. These themes extend previous research by Sutherland and Chur‐Hansen [13], by identifying and contextualising rural skills within the current professional competency requirements. Clinicians entering rural practice via urban trained backgrounds may be less prepared for the challenges of the rural environment, which may impact their longevity in such communities. For example, participants in the current study identified skills such as community integration, workload management and professional development as important to their rural practice. Similar factors, including a sense of community and working conditions (such as workload and opportunities for career development), have been found to impact turnover in rural health practitioners [4].

Participation in rural training has been associated with higher retention rates in rural communities, as it prepares clinicians for rural practice [15]. Though there are limited opportunities for rural psychology placements in Australia [16], standardised approaches and training frameworks are primarily directed towards allied health more broadly [20, 62]. The current study demonstrates that there is a clear need for cadre‐specific support and training for rural psychologists in Australia. In their present form, the identified competencies can be utilised by rural psychologists, supervisors and workplaces to reflect on areas requiring support and direct professional development. However, further research is required to adapt the current findings in a way that better trains, prepares and encourages all psychologists entering or remaining in rural psychology practice. Ultimately, rural communities need more mental health support [1, 3] and ensuring the longevity of the rural psychology workforce is one pathway to improve service access.

### 4.4 Limitations and Future Research

Participant recruitment via professional networks may have introduced self‐selection bias by attracting psychologists with a particular interest in rural psychology experiences and competencies [63]. How participants gained their rural skills/competencies was not directly examined in the current study and it is likely participants' experiences and individualised career trajectories impacted responses. Participant demographic information was limited, and geographic location and employment background, including areas of practice endorsement, may have impacted participants' perspectives. For example, competencies related to leadership or managerial skills were not identified during analysis, likely due to the participant sample and interview questions used. To this end, the small sample used, as well as the specific context, means that the competencies identified by participants may not be generalisable to all rural psychologists [64]. Rather, results may support the transferability of the thematic competencies—that is, their applicability to individuals not directly observed [65]—through further research or policy implementation in other rural psychology contexts. Future research should consider expanding the results of the current study by validating these skills and competencies across a broader rural psychology sample. Likewise, the focus of the current study was limited to identifying the competencies and experiences of rural psychologists, rather than how these competencies were developed, operationalised or supported at organisational or educational levels. Understanding how psychologists learn and identify their rural‐specific competencies is an important next step in future research, particularly in exploring rural workforce development strategies.

## 5 Conclusion

The current study built on limited previous research [13] to identify a broad range of competencies used by rural psychologists. Participants reported the various skills required to manage the unique challenges of rural psychology practice. Though many competencies were consistent with both the broader psychology profession and rural health disciplines more generally, themes such as ethics and risk management suggested there is specificity needed when considering the rural psychology workforce. Future research can expand on these results to inform rural psychology practice and education strategies in order to improve service access to rural communities.

## Author Contributions

**Margot P. Moody:** conceptualization, investigation, funding acquisition, writing – original draft, methodology, writing – review and editing, formal analysis. **Natasha M. Loi:** supervision, writing – review and editing, conceptualization, formal analysis. **Kim J. Usher:** supervision, writing – review and editing. **Adam J. Rock:** supervision, writing – review and editing. **Kylie Rice:** supervision, writing – review and editing, conceptualization, formal analysis, project administration.

## Funding

This work was supported by the Australian Government.

## Conflicts of Interest

The authors declare no conflicts of interest.

## Data Availability

The data that support the findings of this study may be made available from the corresponding author upon reasonable request.
